# Loneliness Trajectories and Dementia Risk Among Mexican American Older Adults: A Latent Growth Model Approach

**DOI:** 10.1093/geroni/igaf122.1761

**Published:** 2025-12-31

**Authors:** Suyoung Kim

**Affiliations:** The University of Chicago, Chicago, Illinois, United States

## Abstract

**Objective:**

Loneliness is an important yet understudied psychosocial determinant of health among Mexican American older adults. This study examines whether loneliness trajectories predict dementia risk in this population, considering the role of functional ability, socioeconomic factors, and psychological well-being.

**Method:**

Data were drawn from Waves 7, 8, and 9 of the Hispanic Established Populations for the Epidemiologic Study of the Elderly (HEPESE). A latent growth model was used to estimate baseline loneliness and its change over time. Predictors included marital status, living arrangement, Instrumental Activities of Daily Living (IADL) limitations, financial strain, and depressive symptoms. Dementia status was assessed at Wave 9 using the Mini-Mental State Examination (MMSE).

**Results:**

On average, loneliness was low at baseline but increased over time. Living alone and depressive symptoms were associated with higher initial loneliness, while IADL limitations and financial strain predicted steeper increases. Both higher baseline loneliness and greater increases over time were linked to a higher risk of dementia at Wave 9, even after adjusting for covariates.

**Discussion:**

These findings suggest that loneliness trajectories contribute to dementia risk among Mexican American older adults. Addressing loneliness, particularly among individuals living alone or experiencing financial and functional difficulties, may be key to mitigating cognitive decline in this population.

